# Supplementary material for: A functional vulnerability framework for biodiversity conservation
Source: Nat Commun. 2022 Sep 1;13:4774. doi: 10.1038/s41467-022-32331-y (PMC9437092; doi:10.1038/s41467-022-32331-y)
Supplement: Supplementary file 1 — Supplementary information [file 41467_2022_32331_MOESM1_ESM.pdf]

Supplementary information

A functional vulnerability framework for biodiversity conservation

Arnaud Auber[1], Conor Waldock[2,3], Anthony Maire[4], Eric Goberville[5], Camille Albouy[6,7], Adam C. Algar[8], Matthew McLean[9], Anik Brind'Amour[10], Alison L. Green[11], Mark Tupper[12,13], Laurent Vigliola[14], Kristin Kaschner[15], Kathleen Kesner-Reyes[16], Maria Beger[17,18], Jerry Tjiputra[19], Aurèle Toussaint[20], Cyrille Violle[21], Nicolas Mouquet[22,23], Wilfried Thuiller[24], David Mouillot[23,25]

Supplementary Methods: Species Distribution Modelling

Reef and coastal grid system
We first defined a coastal and reef grid to identify potentially valid areas for mapping species distributions at a 0.25° by 0.25° scale. Our general approach was to consider locations within a 0.5° buffer of coastal polygons, coral reef polygons, and local SCUBA surveys as valid for shallow-water reef fish presences. For global coastlines we used the Global Self-consistent, Hierarchical, High-resolution Geography dataset (GSHHG) using low resolution level 1 (version 2.3.7)[1]. Additionally, to avoid missing coastal grid cells we inverse buffered this dataset by 0.26°. For coral reef locations we used the UNEP-WCMC "Global distribution of warm-water coral reefs" dataset (version 3)[2]. Finally, for local SCUBA surveys known to be performed in shallow water systems we used a compilation 5,871 SCUBA transects (described below).

Occurrence records and species ranges
We obtained 12,629,945 records for 4,551 species using the AquaMaps occurrence record database (mainly comprised of OBIS, GBIF; personal communication K. Kaschner) (Figure 12). We removed 3,186 fossil records, 6,277,227 duplicated geographic species records, and 442,050 open water and land records that were not consistent with our coastal and coral reef grid. We combined this open-access presence only dataset with in-hand reef fish underwater visual census observations. The in-hand data come from curated citizen science and professional surveys, namely the *Reef Life Survey*[3], *Socio-Ecological Reef Fish dataset (SERF)*[4,5] and *GASPAR* project dataset[6] (see supporting information in Barneche *et al.* 2018 for full sampling description). These contained 253,834 records for 2,758 of the species present in the occurrence dataset at 5,871 unique locations. We filtered to include only unique locations to avoid overfitting resampled locations[7], and removed species with <50 records in unique cells on a 0.25° global reef and coastal grid[8,9]. Our final set comprised of 2,340 species at 496,309 unique georeferenced locations in 20,450 grid cells for analyses. We aligned all submitted taxonomic names across data sources using the function validate_names in rfishbase[10] (version 3.0.4).

Environmental variables
We modelled species distributions and abundances using 6 environmental variables commonly used for modelling marine species[11,12,13]: maximum and minimum sea surface temperature (SST), minimum sea surface salinity (SSS), mean net primary productivity (NPP), minimum pH, mean degree heating weeks (DHW) and human gravity in a 500km^2 area. Being ectothermic, fishes have strong metabolic and physiological responses to sea surface temperature. As such, occupancy limits of sea surface temperature correlate well with species thermal physiological limits[14], there is correspondence amongst physiological and abundance optima[15,16] and temperature also predicts well range shifts[17,18] and temporal change in fish assemblages[19,20]. Salinity and pH have direct effects on reef fish osmotic and ionic balance[21,22] and can indirectly affect reef fish distributions by reducing the quality, amount and diversity of calcareous reef habitat[23,24,18]. We chose minimum salinity because of the reef fish assemblage diversity and biogeography are affected by freshwater flows[25,26,27] and minimum pH because of the direct and indirect effects of ocean acidification on fishes[28,29]. Primary productivity indicates the amount of phytoplankton that form the base of many marine food chains, we included mean annual NPP as an indicator of annual energy available to upper trophic levels. Degree-heating weeks represent the accumulated thermal stress and is a strong predictor of coral bleaching[30,31,32]. Degree heating weeks is expressed as the unit °C-weeks. The daily degree heating week is calculated as the sum of temperatures above 1°C of summertime maximum temperatures for 84 days (12 weeks) prior, divided by 7 to give a weekly value[31]. Finally, we included the gravity of human impacts to control for the effect of human population on observation of species through introducing sampling biases[33,34] or negatively affecting species' populations[35,5]. We ensured our models were robust to variable multicollinearity which can bias range projections under climate change[36]. For each model, we checked that the Pearson correlation between pairs of variables were <0.7, and if not, retained the variable with the highest deviance explained and lowest error rate (depending on the model algorithm, see below). We iterated this process until we obtained a set of uncorrelated and maximally explanatory variables.

Environmental background data
The vast majority of our occurrence records come from unstructured surveys where species absences are not recorded. As such, we generated background environmental data (pseudo-absences) using a target-group approach[37]. Target group sampling was expected to reduce bias in our habitat suitability estimates by sampling background data from the available set of occurrence records. This process gave the background data a similar bias to the presence data and helped avoid extrapolation of absences beyond sampled environmental space. This approach has been demonstrated as robust in previous large-scale analyses of marine species distributions[38]. We assumed all our reef species form an appropriate target group because similar sampling tools are used to generate occurrence records (e.g., SCUBA underwater visual surveys). We generated ten times the number of background points compared to presence points[39], sub-sampling with replacement when too few background points exist. To constrain the selection of background data to be within species potential ranges, we first produced a convex hull around species presences, retained intersecting marine provinces, and identified target record within this province range. We sampled 50% of background points from Spalding marine provinces that contain species presences, and 50% from adjacent provinces with no observed presences. The background data therefore balances our capacity to resolve internal range structure in addition to range edges[40]. To ensure models were robust to stochastic background data generation, we repeated the background data generation five times and refitted all models for each background sample.

Fitting occurrence-based species distribution models
We used a set of algorithms that represent a gradient in environmental response complexity (generalized linear models: GLM, generalized additive models: GAM, and random forests: RF). The chosen statistical algorithm is an important source of variation in species distribution model predictions[41], particularly under climate change[42,36]. We fitted GLM and GAM using binomial error distributions and RFs as classification trees.

We fitted GLMs with a binomial error distribution using least squares. We ensured models converged using ordered backward stepwise model selection based on model term contributions, which ensures a model converges before performing backward selection based on likelihood-ratio tests. Our GLMs represent the simplest response shapes of our model set so we fitted linear and quadratic terms only. We fitted GLMs in the *stats* R package after the optimal model structure was identified using a combination of packages *buildmer*[43] and *glmmTMB*[44] which provided efficient tools for model fitting and selection.

We fitted GAMS with a binomial error distribution using quadratically penalized likelihood maximisation with the package *mgcv*[45,46]. We first tested that each independent covariate produces an identifiable model and included valid covariates in multiple regressions. We performed model selection using null-space penalization to reduce the effect of non-important covariates to 0 (i.e., setting the argument select = T)[47]. We used thin plate regressions splines and a basis dimension of 5 to allow intermediate flexibility and computational efficiency[46].

We fitted random forests using the R package *randomForest*[48]. Hyperparameter selection can have a large influence on model fit and default settings are unlikely to be optimal[49]. We tuned the number of variables randomly sampled at each split (*mtry*) and size of the sample drawn for each tree (*sampsize*). We used a grid approach to hyperparameter selection by varying *mtry* between the square root of the number of covariates, as suggested in (Breiman 2001)[50], and the maximum number of covariates. We varied *sampsize* from 10% to 100% of the training sample in 10% increments. We balanced *sampsize* proportionate to the representation of classification groups (0,1) in the testing set using the *strata* setting. Because we varied the sample size, the optimal *nodesize* parameter is uninfluential and we set it to 1[49]. We evaluated the performance of the random forest hyperparameter grid using the true-skill statistic (TSS) of predicted vs. observed occurrence, and repeated the above steps 5 times to obtain a mean TSS due to the stochastic nature of the RF algorithm[51]. We fitted our final RF with the optimal hyperparameters that maximised TSS.

Spatial cross-validation for model evaluation
In evaluating model performance, spatial similarity can positively bias model evaluations. Within each background data set (n=5) we created a 5-fold spatially blocked cross-validation testing datasets (within each 5-fold pseudo-absence repetition) that were independent from training data. We used the *spatialBlock* function in the R package '*blockCV*'[52] with systematic block selection optimised over 500 iterations. We set the area of blocks as 10% of species occurrence area, determined as a bounding box of occurrences, such that each species blocking system was proportional to species' range size. For occurrence-based models, we balanced folds according to the ratio of species occurrences and background data. We compared predicted habitat suitability to held-out presences and pseudo-absence data (Figure S13). We estimated the mean model true-positive rate (sensitivity), true-negative rate (specificity), true skill statistic (TSS), area under the curve statistic (AUC) using *ecospat* package in R[53]. In addition, for GLM and GAM we estimated model deviance squared (D^2) as a measure of variation explained, for RF we also estimated model error rate as the proportion of combined false negatives and false positives. We estimated model predictive bias as an indicator of model overfitting. To do so, we evaluated the R^2 between global model predictions (i.e., those used in the final analysis) and cross validation predictions (i.e., those used to evaluate our model performance). High model predictive bias (low R^2) indicates model overfitting, whereby models fitted using all data are expected to poorly predict habitat suitability in new areas.

Model projections
We predicted habitat suitability for the present day (1985-2015) across our coastal and coral reef grid. We estimated habitat suitability under future climate conditions derived from six fully coupled global circulation models under the Coupled Model Inter-comparison Project Phase 6 (CMIP6): CNRM-ESM2-1, GFDL-ESM4, IPSL-CM6A-LR, MIROC-ES2L, MPI-ESM1-2-LR, NorESM2-MM, UKESM1-0-LL. We selected models that provided SST, SSS, pH and NPP and forced our models under changes in these four variables. To match our present-day environmental variables used for building species distribution models, we averaged mean, minimum and maximum monthly values to yearly averages, and then to climatological averages between 1985-2015, 2015-2040, 2041-2070 and 2071-2100. We used climatological averages to remove the influence of decadal climate oscillations (e.g., El Niño Southern Oscillation, Pacific Decadal Oscillation). We considered two Shared Socioeconomic Pathways: SSP1-2.6 and SSP5-8.5 which representative of high ('fossil fuel development') and low ('sustainable') emission futures where anthropogenic greenhouse gas emissions induce $2.6Wm^{-2}$ and $8.5Wm^{-2}$ increase in radiative forcing by the year 2100[54], approximating Representative Concentration Pathway 2.6 and 8.5 in CMIP5. We estimated the change in each variable from 1985-2015 compared to future time periods, and applied this change to our present day spatially gridded environmental data.

We predicted species distributions under future conditions for each species. To account for uncertainty arising from different model assumptions, we made a separate prediction for background data iterations (n=5 for distributions), earth systems models (n=6), shared socioeconomic pathways (n=2). To ensure predictions of suitability were robust, we first removed all models with an out-of-sample TSS < 0.35[38]. Across model algorithms and background data iterations, we estimated the mean and standard deviation of suitability predictions weighted by the TSS. Next, we estimated the mean and standard deviation of habitat suitability predictions across all earth system model projections, before constraining future predictions by dispersal scenarios (see below).

Dispersal
Dispersal rates are poorly quantified and unknown for many fish species, especially at the relatively short time-scales considered here. Therefore, we modelled a range of dispersal scenarios (as in Warren *et al.* 2018[55]). First, we assumed no dispersal, in each new time-step species were constrained to their range in the previous time-step. In this scenario, extirpations from grid cells could not be recovered, and range expansions cannot occur. Second, we modelled dispersal informed by literature derived realized range shifts. Lenoir et al. (2020)[56] report an mean latitudinal shift of 5.95±0.94km per year in fishes, Sunday *et al.* (2015)[57] report shifts of 3.8±7 km per year, with larger ranged species tracking at a rate of 9.2km per year, and Fredston-Hermann *et al.* (2020)[58] report shifts of cold range edges of 6.7±0.8km per year but non-significant shifts in warm range edges. We modelled range shifts potential

of 10km per year which is greater than the mean for most estimates because i) literature derived values often only consider north-south movements distances (e.g., Lenoir *et al.* 2020[56]), ii) depth shifts are unlikely to occur for many reef species considered here[59] but often occur for the species considered in studies of realized range shifts, thereby literature estimates are likely to underestimate realized range shifts[17,19]. We did not consider dispersal differences amongst specific families or trait sets because evidence for strong trait-based effects on range shift magnitudes is mixed[60]. In fishes, Pinsky et al. (2013)[17] find marine species capacity to track shifting climate isotherms are very weakly related to species traits because multiple different strategies to facilitate dispersal exist (e.g., adult movement, larval dispersal; but see Sunday *et al.* 2015[57]). Our third scenario was a ‘no-limitation’ scenario whereby species could migrate into all climatically suitable cells in their native province and additional adjacent provinces.

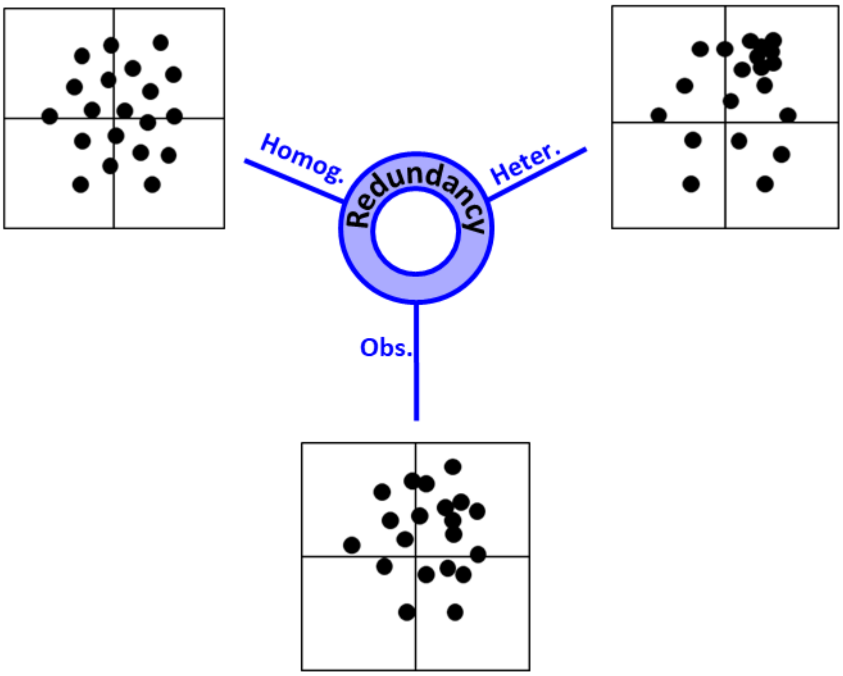

Figure 1. Trait spaces (PCoA biplots) of the 2 pseudo-communities and the observed community in cases where data are presence/absence only. The blue circle separates pseudo-communities into 3 main categories according to the distribution of functional redundancy (heterogeneous: 'Heter.'; homogeneous: 'Homog.'; observed: 'Obs.').

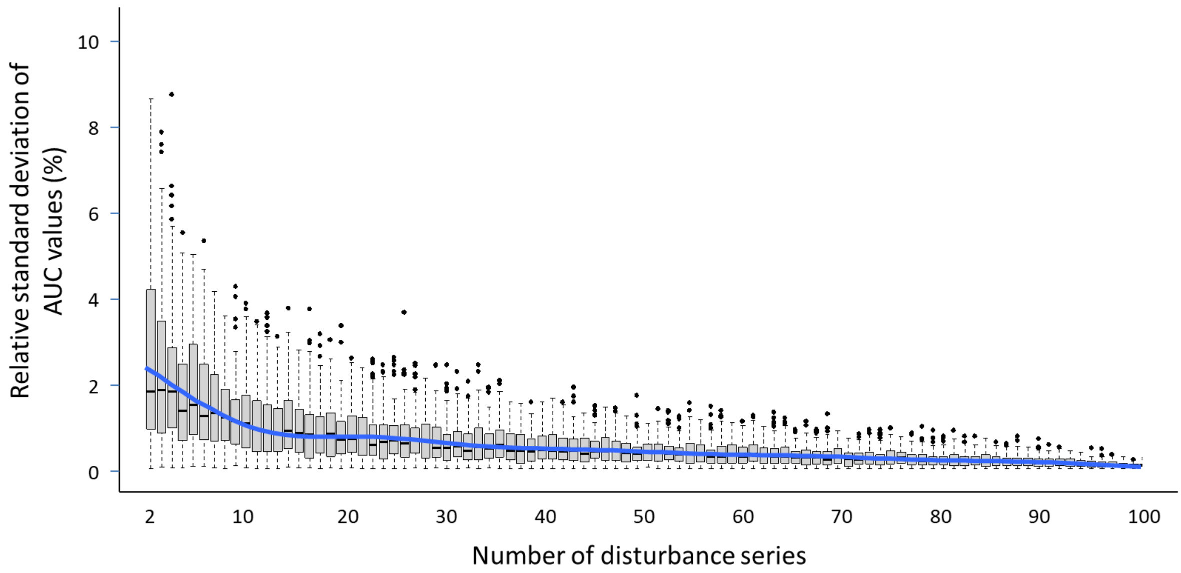

Figure 2. Sensitivity of AUC values to the number of disturbance series applied on communities. To test the effect of the number of disturbance series, 99 rarefaction curves were performed among which a varying number of curves (from 2 to 99) were randomly selected 100 times (n=[99-1]*100=9800 rarefaction curves). Boxes are defines by lower and upper box boundaries 25th and 75th percentiles, respectively, median is defined by the line inside box. Lower and upper error lines correspond to lower quartile minus 1.5 times interquartile range (IQR) and upper quartile plus 1.5 times IQR, respectively. Points falling outside correspond to minimal and maximal values.

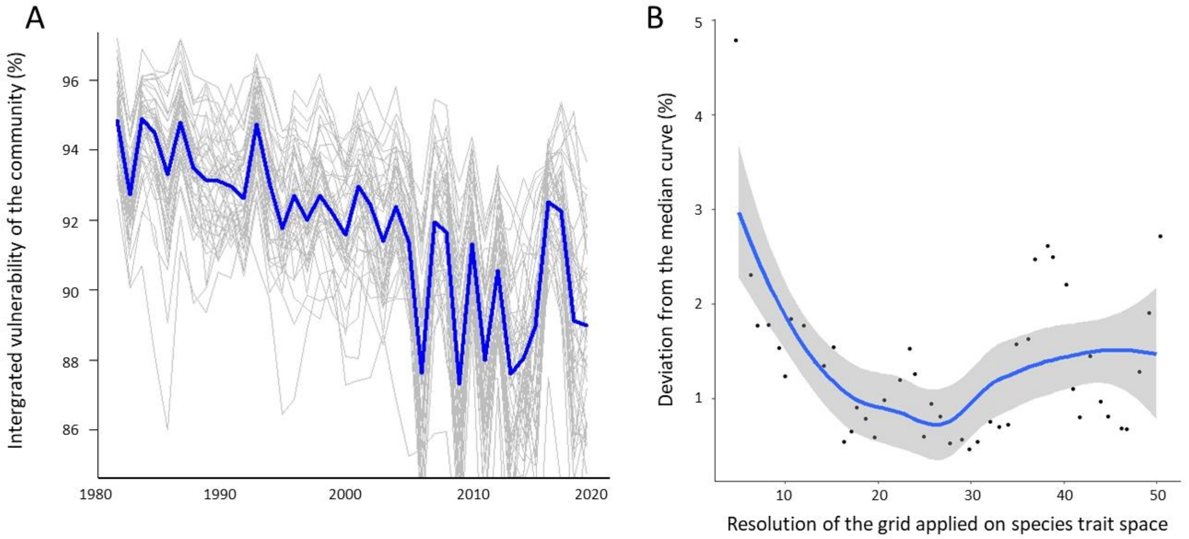

Figure 3. Sensitivity of the vulnerability index to the grid resolution applied on the species trait space (applied on the North Sea fish community case study). **A**. Temporal dynamics of the vulnerability under various grid resolution (the blue line is constructed from to the median value each year; grey lines corresponds to vulnerability values computed from 5*5 to 50*50 cells). **B**. Mean difference between the vulnerability computed with a given grid resolution and the reference vulnerability values (i.e. blue curve in Figure S3.A.). Data are presented as loess predicted values ± 5(and 10)*standard error. Data are presented as loess predicted values ± 95% confidence interval.

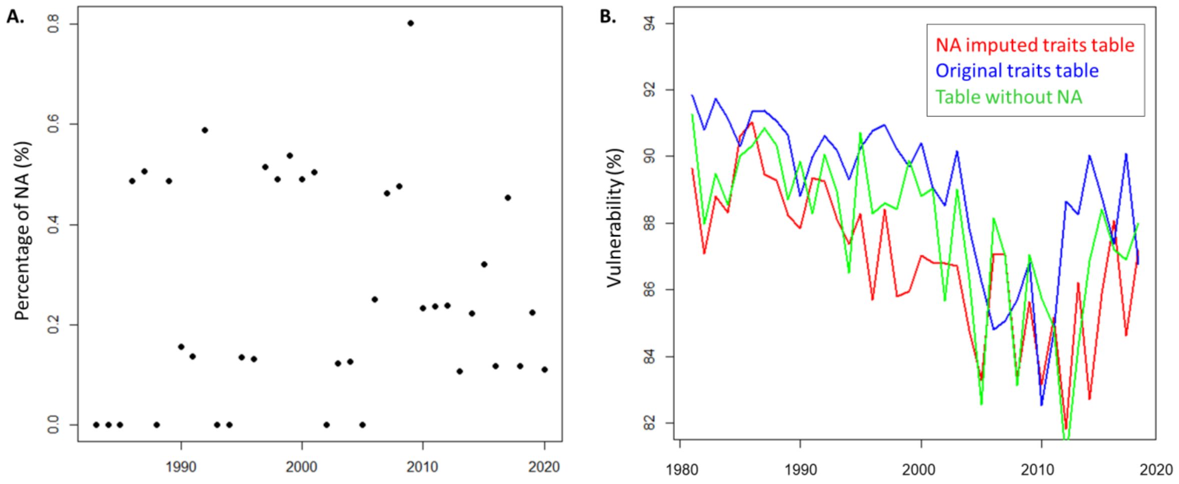

Figure 4. Effect of 'Not Available' (NA) data in species traits tables on final vulnerability values for the North Sea fishes case study. **A**. Temporal trend in the percentage of NA in species traits tables. **B**. Temporal trends in vulnerability values computed from the original traits table (i.e., with all NA considered; blue line), from the NA imputed traits table (red line) and from the traits table where all species with at least one NA were removed from analyses (green line).

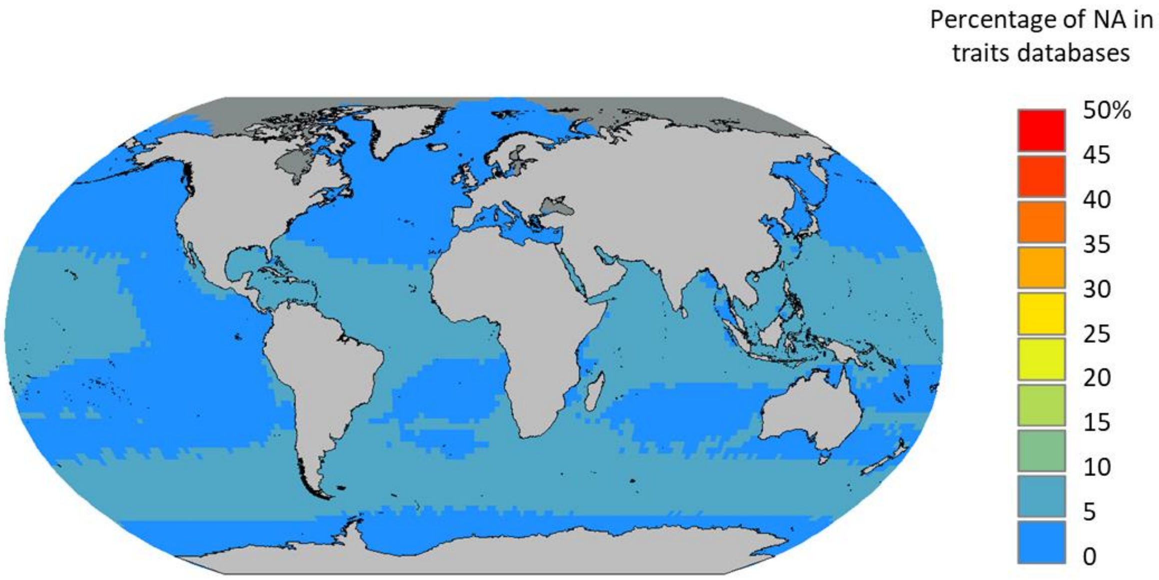

Figure 5. Spatial distribution of NA in marine mammals traits dataset. Background map shapefiles are available on the NOAA website: https://www.ngdc.noaa.gov/mgg/shorelines/data/gshhg/latest/.

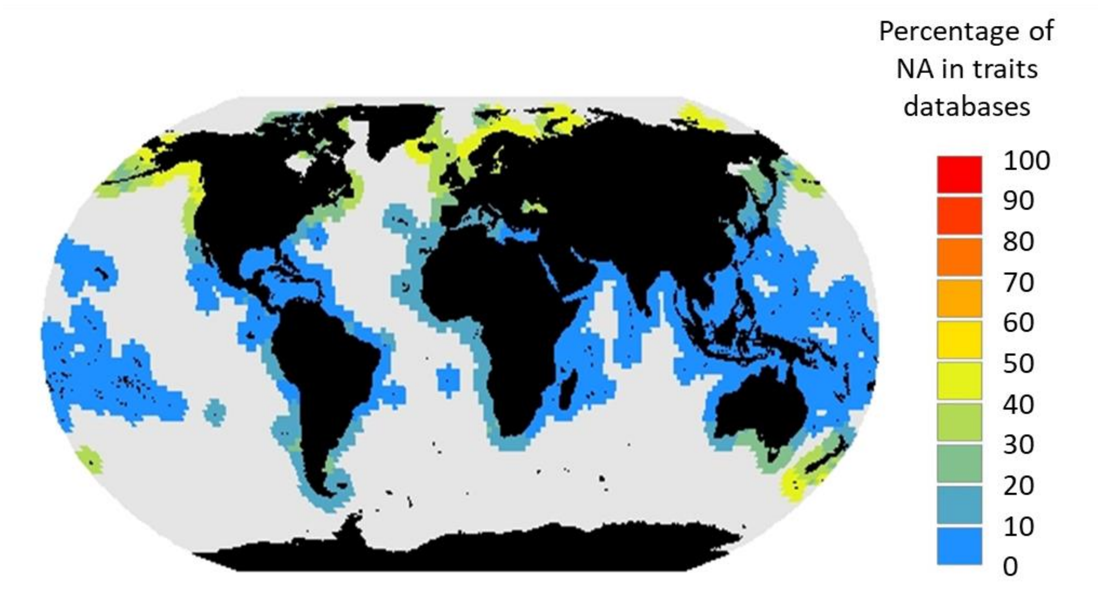

Figure 6. Spatial distribution of NA in global scale reef fishes traits dataset. Background map shapefiles are available on the NOAA website: https://www.ngdc.noaa.gov/mgg/shorelines/data/gshhg/latest/.

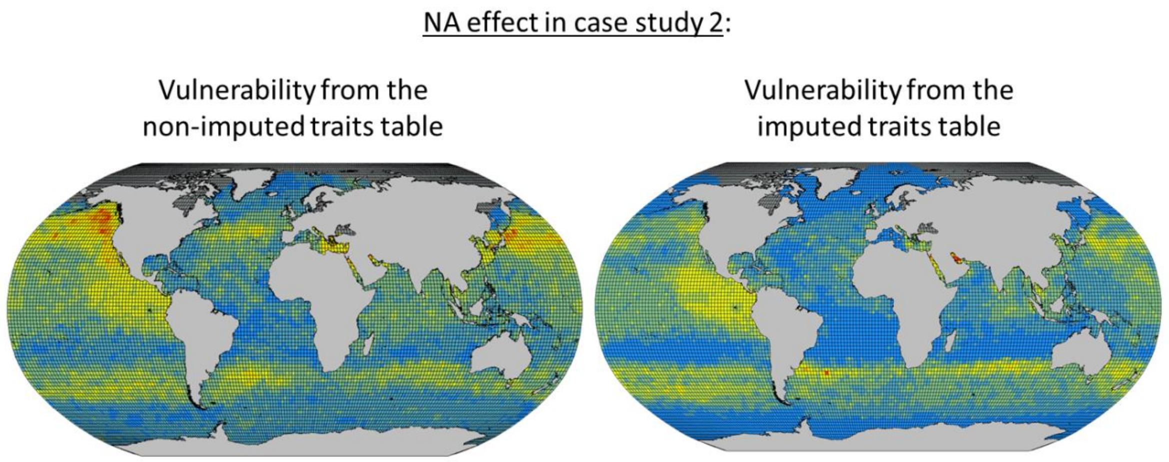

Figure 7. Effects of non available data in species traits dataset on vulnerability values of the marine mammals’s communities. Background map shapefiles are available on the NOAA website: https://www.ngdc.noaa.gov/mgg/shorelines/data/gshhg/latest/.

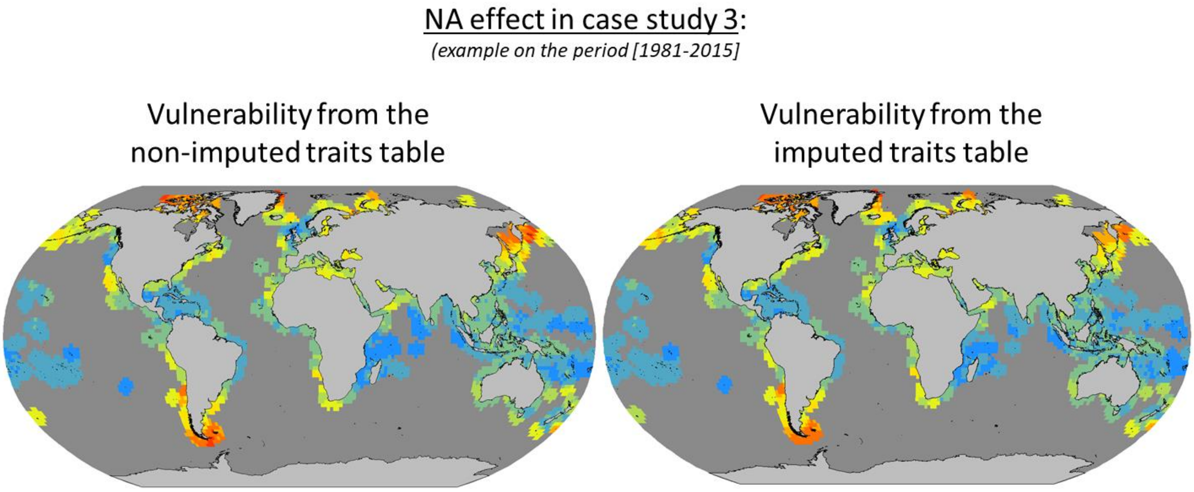

Figure 8. Effects of non available data in species traits dataset on vulnerability values of the global reef fish communities. Background map shapefiles are available on the NOAA website: https://www.ngdc.noaa.gov/mgg/shorelines/data/gshhg/latest/.

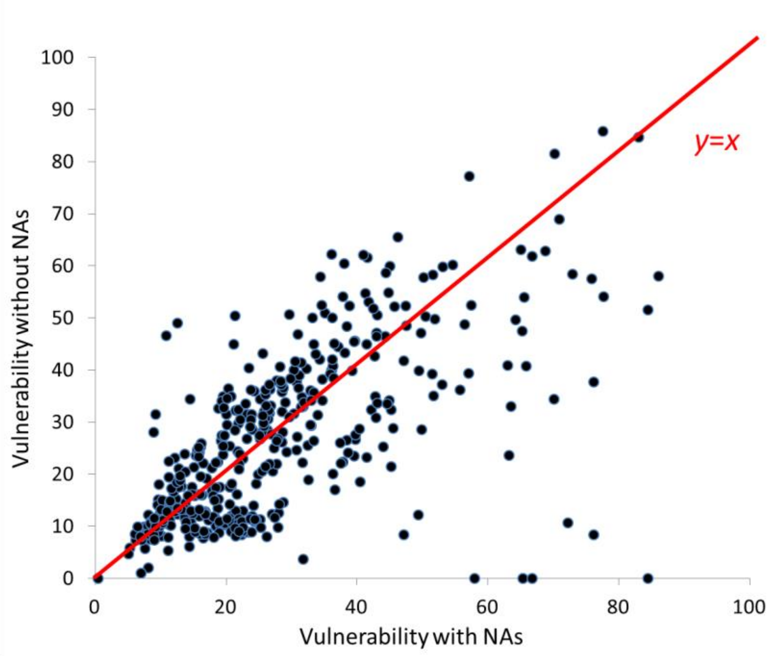

Figure 9. Correlation between vulnerability computed from the original traits table (i.e., with all NA considered) and vulnerability computed from the NA imputed traits table for the global scale reef fishes case study (*r* = 0.67).

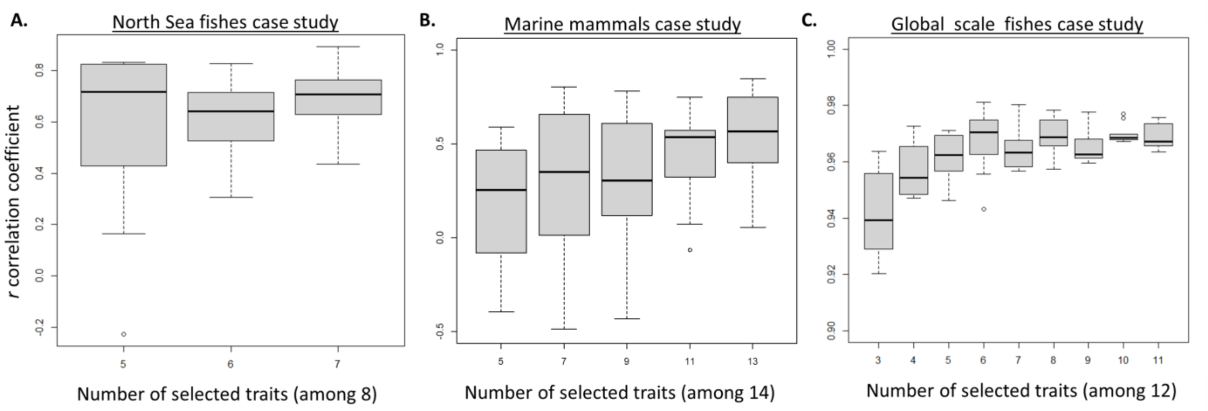

Figure 10. Effect of species trait deletion on vulnerability results. Pearson's correlation coefficient between reference vulnerability values (i.e. computed from all traits) and vulnerability values computed from subsetted traits tables for the three study cases: **A**. North Sea fishes, **B**. Marine mammals, and **C**. global scale reef fishes. Boxes are defines by lower and upper box boundaries 25th and 75th percentiles, respectively, median is defined by the line inside box. Lower and upper error lines correspond to lower quartile minus 1.5 times interquartile range (IQR) and upper quartile plus 1.5 times IQR, respectively. Points falling outside correspond to minimal and maximal values.

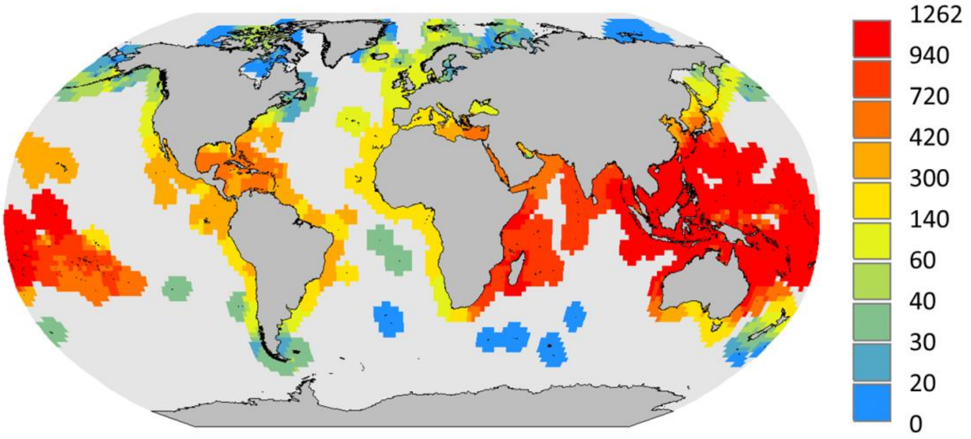

Figure 11. Global species richness of coastal fish communities at the horizon [2041-2070]. Background map shapefiles are available on the NOAA website: https://www.ngdc.noaa.gov/mgg/shorelines/data/gshhg/latest/

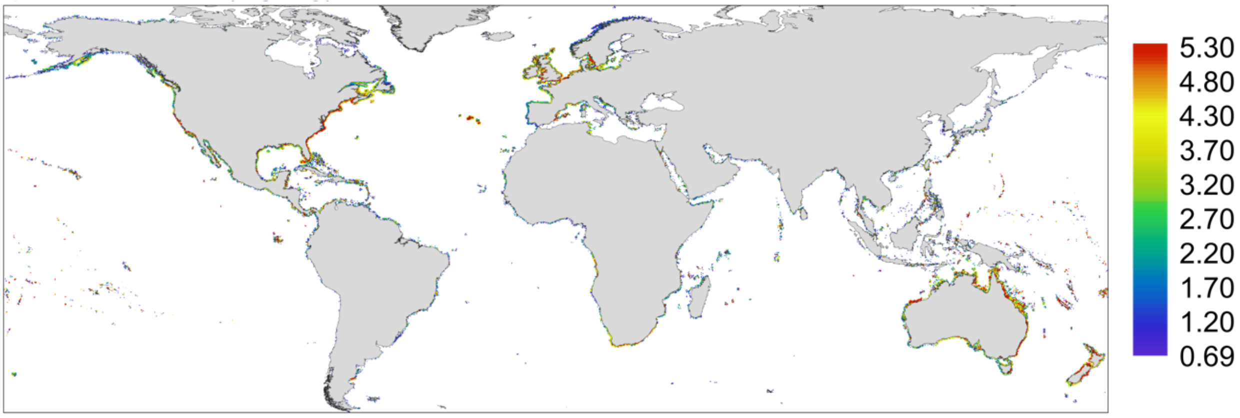

Figure 12. Spatial map of occurrence records used to build reef fish species distribution models. Background map shapefiles are available on the NOAA website: https://www.ngdc.noaa.gov/mgg/shorelines/data/gshhg/latest/

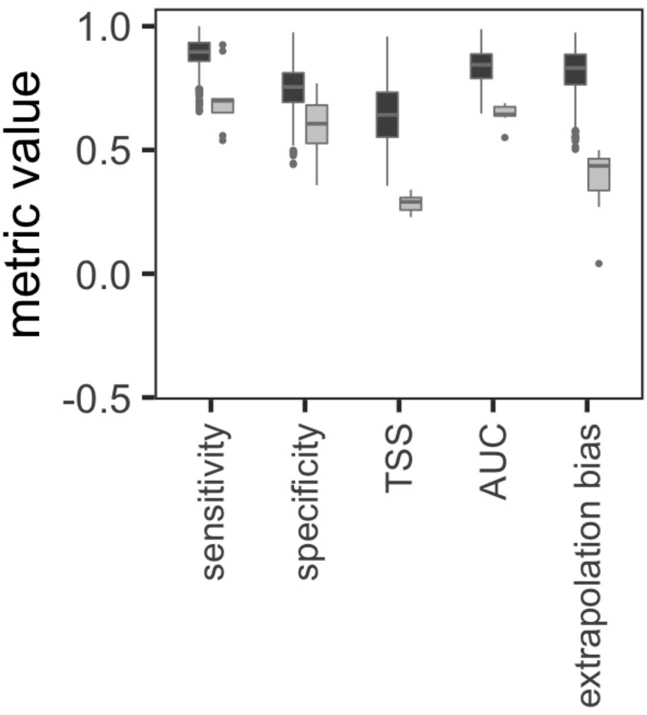

Figure 13. Evaluation summaries of species distribution models for occurrence-based SDMs. All metrics indicate out-of-sample spatially blocked cross-validations. Dark gray indicates species retained in models, light gray indicates species models fitted but not considered due to low performance. Performance metrics are first mean averaged over cross validation folds (n = 5), next over background pseudo-absences (n = 5), and finally over model algorithms (n = 3). TSS = true skill statistic, AUC = area under the receiver-operator curve. Extrapolation bias is the R^2 between predictions of models calibrated on the full dataset vs. the training set and is an indicator of the % variation explained for novel data points not included in the model. Boxes are defines by lower and upper box boundaries 25th and 75th percentiles, respectively, median is defined by the line inside box. Lower and upper error lines correspond to lower quartile minus 1.5 times interquartile range (IQR) and upper quartile plus 1.5 times IQR, respectively. Points falling outside correspond to minimal and maximal values.

Supplementary References

1. Wessel, P. & Smith, W. H. F. A global, self-consistent, hierarchical, high-resolution shoreline database. *J. Geophys. Res. B Solid Earth*, **101**, 8741–8743 (1996).
2. UNEP-WCMC, WFC, WRI & TNC. Global distribution of warm-water coral reefs, compiled from multiple sources including the Millennium Coral Reef Mapping Project. Version 3.0. Includes contributions from IMaRS-USF and IRD (2005), IMaRS-USF (2005) and Spalding et al. (2001). Cambridge Univ. (2010).
3. Edgar, G. J. & Stuart-Smith, R. D. Systematic global assessment of reef fish communities by the Reef Life Survey program. *Sci. Data*, 1, 140007 (2014).
4. Mora, C. et al. Global human footprint on the linkage between biodiversity and ecosystem functioning in reef fishes. *PLoS Biol*., 9 (2011).
5. Cinner, J. E. et al. Gravity of human impacts mediates coral reef conservation gains. *Proc. Natl. Acad. Sci. U. S. A.*, **115**, E6116–E6125 (2018).
6. Barneche, D. R. et al. Body size, reef area and temperature predict global reef-fish species richness across spatial scales. *Glob. Ecol. Biogeogr*., **28**, 315–327 (2018).
7. Boria, R. A., Olson, L. E., Goodman, S. M. & Anderson, R. P. Spatial filtering to reduce sampling bias can improve the performance of ecological niche models. *Ecol. Modell*., **275**, 73–77 (2014).
8. Wisz, M. S. et al. Effects of sample size on the performance of species distribution models. *Divers. Distrib*., **14**, 763–773 (2008).
9. Feeley, K. J. & Silman, M.R. Keep collecting: accurate distribution modelling requires more collections than previously thought. *Divers. Distrib*., **17**, 1132–1140 (2011).
10. Boettiger, C., Lang, D. T. & Wainwright, P. C. rfishbase: exploring, manipulating and visualizing FishBase data from R. *J. Fish Biol.*, **81**, 2030–2039 (2012).
11. Ready, J. et al. Predicting the distributions of marine organisms at the global scale. *Ecol. Modell.*, **221**, 467–478 (2010).
12. Cheung, W. W. L., et al. Structural uncertainty in projecting global fisheries catches under climate change. *Ecol. Modell*., **325**, 57–66 (2016).
13. Asch, R. G., Cheung, W. W. L. & Reygondeau, G. Future marine ecosystem drivers, biodiversity, and fisheries maximum catch potential in Pacific Island countries and territories under climate change. *Mar. Policy*, **88**, 285–294 (2018).
14. Webb, T. J., Lines, A. & Howarth, L. M. Occupancy-derived thermal affinities reflect known physiological thermal limits of marine species. *Ecol. Evol*., **10**, 7050–7061 (2020).
15. Waldock, C., Stuart-Smith, R. D., Edgar, G. J., Bird, T. J. & Bates, A. E. The shape of abundance distributions across temperature gradients in reef fishes. *Ecol. Lett*., **22**, 685–696 (2019).
16. Payne, N. L. et al. Fish heating tolerance scales similarly across individual physiology and populations. *Commun. Biol*., **4**, 264 (2021).
17. Pinsky, M. L., Worm, B., Fogarty, M. J., Sarmiento, J. L. & Levin, S. A. Marine taxa track local climate velocities. *Science*, **341**, 1239–1242 (2013).
18. Sunday, J. M. et al. Ocean acidification can mediate biodiversity shifts by changing biogenic habitat. *Nat. Clim. Chang*., **7**, 81–85 (2017).
19. Burrows, M. T., et al. Ocean community warming responses explained by thermal affinities and temperature gradients. *Nat. Clim. Chang*., **9**, 959–963 (2019).
20. Antão, L. H., et al. Temperature-related biodiversity change across temperate marine and terrestrial systems. *Nat. Ecol. Evol*., **4**, 927–933 (2020).
21. Evans, D. H., Piermarini, P.M. & Choe, K.P. The multifunctional fish gill: Dominant site of gas exchange, osmoregulation, acid-base regulation, and excretion of nitrogenous waste. *Physiol. Rev*., **85**, 97–177 (2005).
22. Evans, D. H. & Claiborne, J. B. Osmotic and Ionic Regulation in Fishes. In: Osmotic and Ionic Regulation: Cells and Animals. pp. 295–366 (2008).
23. Hoegh-Guldberg, O. et al. Coral reefs under rapid climate change and ocean acidification. *Science*, **318**, 1737–42 (2007).
24. Wenger, A. S., et al. Effects of reduced water quality on coral reefs in and out of no-take marine reserves. *Conserv. Biol.*, **30**, 142–153 (2016).
25. Briggs, J. C. & Bowen, B. W. A realignment of marine biogeographic provinces with particular

reference to fish distributions. *J. Biogeogr*., **39**, 12–30 (2012).
26. Neves, L. M., Teixeira-Neves, T. P., Pereira-Filho, G. H. & Araújo, F. G. The farther the better: Effects of multiple environmental variables on reef fish assemblages along a distance gradient from river influences. *PLoS One*, **11**, e0166679 (2016).
27. Mellin, C., et al. Spatial resilience of the Great Barrier Reef under cumulative disturbance impacts. *Glob. Chang. Biol*., **25**, 2431–2445 (2019).
28. Wittmann, A. C. & Pörtner, H. O. Sensitivities of extant animal taxa to ocean acidification. *Nat. Clim. Chang*., **3**, 995–1001 (2013).
29. Heuer, R. M. & Grosell, M. Physiological impacts of elevated carbon dioxide and ocean acidification on fish. *Am. J. Physiol. Integr. Comp. Physiol*., **307**, R1061–R1084 (2014).
30. Glynn, P. W. & D'Croz, L. Experimental evidence for high temperature stress as the cause of El Nino-coincident coral mortality. *Coral Reefs*, **8**, 181–191 (1990).
31. Liu, G., et al. Reef-scale thermal stress monitoring of coral ecosystems: New 5-km global products from NOAA coral reef watch. *Remote Sens*., **6**, 11579–11606 (2014).
32. Hughes, T. P. et al. Global warming and recurrent mass bleaching of corals. *Nature*, **543**, 373–377 (2017).
33. Tessarolo, G., Rangel, T. F., Araújo, M. B. & Hortal, J. Uncertainty associated with survey design in species distribution models. *Divers. Distrib*., **20**, 1258–1269 (2014).
34. Yang, W., Ma, K. & Kreft, H. Environmental and socio-economic factors shaping the geography of floristic collections in China. *Glob. Ecol. Biogeogr*., **23**, 1284–1292 (2014).
35. Maire, E. et al. How accessible are coral reefs to people? A global assessment based on travel time. *Ecol. Lett*., **19**, 351–360 (2016).
36. Brun, P. et al. Model complexity affects species distribution projections under climate change. *J. Biogeogr*., 1–13 (2019).
37. Phillips, S. J. et al. Sample selection bias and presence-only distribution models: Implications for background and pseudo-absence data. *Ecol. Appl*., **19**, 181–197 (2009).
38. Righetti, D., Vogt, M., Gruber, N., Psomas, A. & Zimmermann, N. E. Global pattern of phytoplankton diversity driven by temperature and environmental variability. *Sci. Adv*., **5**, 1–11 (2019).
39. Barbet-Massin, M., Jiguet, F., Albert, C. H. & Thuiller, W. Selecting pseudo-absences for species distribution models: How, where and how many? *Methods Ecol. Evol*., **3**, 327–338 (2012).
40. VanDerWal, J., Shoo, L. P., Graham, C. & Williams, S. E. Selecting pseudo-absence data for presence-only distribution modeling: How far should you stray from what you know? *Ecol. Modell*., **220**, 589–594 (2009).
41. Merow, C. et al. What do we gain from simplicity versus complexity in species distribution models? *Ecography* (Cop.)., **37**, 1267–1281 (2014).
42. Buisson, L., Thuiller, W., Casajus, N., Lek, S. & Grenouillet, G. Uncertainty in ensemble forecasting of species distribution. *Glob. Chang. Biol*., **16**, 1145–1157 (2010).
43. Voeten, C. C. buildmer: Stepwise Elimination and Term Reordering for Mixed-Effects Regression (2020).
44. Brooks, M. E. et al. glmmTMB Balances Speed and Flexibility Among Packages for Zero-inflated Generalized Linear Mixed Modeling. *R J*., **9**, 378–400 (2017).
45. Wood, S. Fast stable restricted maximum likelihood and marginal likelihood estimation of semiparametric generalized linear models. J. R. Stat. Soc. Ser. B (Statistical Methodol.), **73**, 3–36 (2011).
46. Wood, S. Generalized Additive Models: An Introduction with R. 2nd edn. Chapman and Hall/CRC (2017).
47. Marra, G. & Wood, S. N. Practical variable selection for generalized additive models. *Comput. Stat. Data Anal*., **55**, 2372–2387 (2011).
48. Liaw, A. & Wiener, M. Classification and Regression by randomForest. *R News*, **3**, 18–22 (2002).
49. Scornet, E. Tuning parameters in random forests. *ESAIM Proc. Surv*., **60**, 144–162 (2017).
50. Breiman, L. Random Forests. *Mach. Learn*., **45**, 5–32 (2001).
51. Allouche, O., Tsoar, A. & Kadmon, R. Assessing the accuracy of species distribution models: Prevalence, kappa and the true skill statistic (TSS). *J. Appl. Ecol*., **43**, 1223–1232 (2006).
52. Valavi, R., Elith, J., Lahoz-Monfort, J. J. & Guillera-Arroita, G. BLOCKCV: An R package for

generating spatially or environmentally separated folds for k -fold cross-validation of species distribution models. *Methods Ecol. Evol.*, **10**, 225–232 (2019).
53. Broennimann, O., Cola, V. Di & Guisan, A. ecospat: Spatial Ecology Miscellaneous Methods. R package version 3.1. https://CRAN.R-project.org/package=ecospat (2020).
54. Riahi, K. et al. The Shared Socioeconomic Pathways and their energy, land use, and greenhouse gas emissions implications: An overview. *Glob. Environ. Chang.*, **42**, 153–168 (2017).
55. Warren, R., Price, J., Graham, E., Forstenhaeusler, N. & VanDerWal, J. The projected effect on insects, vertebrates, and plants of limiting global warming to 1.5°C rather than 2°C. *Science*, **360**, 791–795 (2018).
56. Lenoir, J. et al. Species better track climate warming in the oceans than on land. *Nat. Ecol. Evol.*, **4**, 1044–1059 (2020).
57. Sunday, J. M. et al. Species traits and climate velocity explain geographic range shifts in an ocean-warming hotspot. *Ecol. Lett.*, **18**, 944–953 (2015).
58. Fredston-Hermann, A., Selden, R., Pinsky, M., Gaines, S. D. & Halpern, B. S. Cold range edges of marine fishes track climate change better than warm edges. *Glob. Chang. Biol.*, **26**, 2908–2922 (2020).
59. Rocha, L. A. et al. Mesophotic coral ecosystems are threatened and ecologically distinct from shallow water reefs. *Science*, **361**, 281–284 (2018).
60. Beissinger, S. R. & Riddell, E. A. Why Are Species' Traits Weak Predictors of Range Shifts? *Annu. Rev. Ecol. Evol. Syst.*, **52**, 47–66 (2021).
