# Supplementary material for: A functional vulnerability framework for biodiversity conservation
Source: Nat Commun. 2022 Sep 1;13:4774. doi: 10.1038/s41467-022-32331-y (PMC9437092; doi:10.1038/s41467-022-32331-y)
Supplement: Supplementary file 3 — Reporting Summary [file 41467_2022_32331_MOESM3_ESM.pdf]

nature portfolio

Corresponding author(s): Arnaud Auber

Last updated by author(s): Jun 24, 2022

Reporting Summary

Nature Portfolio wishes to improve the reproducibility of the work that we publish. This form provides structure for consistency and transparency in reporting. For further information on Nature Portfolio policies, see our Editorial Policies and the Editorial Policy Checklist.

Statistics

For all statistical analyses, confirm that the following items are present in the figure legend, table legend, main text, or Methods section.

n/a	Confirmed	
☐	☒	The exact sample size (*n*) for each experimental group/condition, given as a discrete number and unit of measurement
☐	☒	A statement on whether measurements were taken from distinct samples or whether the same sample was measured repeatedly
☐	☒	The statistical test(s) used AND whether they are one- or two-sided<br>*Only common tests should be described solely by name; describe more complex techniques in the Methods section.*
☐	☒	A description of all covariates tested
☐	☒	A description of any assumptions or corrections, such as tests of normality and adjustment for multiple comparisons
☐	☒	A full description of the statistical parameters including central tendency (e.g. means) or other basic estimates (e.g. regression coefficient) AND variation (e.g. standard deviation) or associated estimates of uncertainty (e.g. confidence intervals)
☐	☒	For null hypothesis testing, the test statistic (e.g. *F*, *t*, *r*) with confidence intervals, effect sizes, degrees of freedom and *P* value noted<br>*Give P values as exact values whenever suitable.*
☒	☐	For Bayesian analysis, information on the choice of priors and Markov chain Monte Carlo settings
☒	☐	For hierarchical and complex designs, identification of the appropriate level for tests and full reporting of outcomes
☐	☒	Estimates of effect sizes (e.g. Cohen's *d*, Pearson's *r*), indicating how they were calculated

Our web collection on statistics for biologists contains articles on many of the points above.

Software and code

Policy information about availability of computer code

Data collection	R was used to extract data of case study 1 (through the getDATRAS function in the icesDatras package).
Data analysis	All statistical analyses were performed under the R version 4.1.0 (R Core Team, 2021).<br><br>We used R packages: funrar package (v.1.4.1) for computation of functional distinctiveness, ape package (v.5.5) to perform Principal Coordinates Analyses, maps package (v.3.3.0) to build the map in figure 2A, and missForest package (v.1.4) to impute NA in species traits tables (supplementary analyses). In Supplemntary information : stats (v.4.1.0), buildmer (v.2.5), glmTMB(v.1.1.3), mgcv (v1.8.35), randomForest (v.4.7-1.1), blockCV (v.2.1.4), ecospat (v3.3).<br><br>Codes to run vulnerability analyses as been made available at:<br>Case study 1: https://figshare.com/s/9d3cd1d6f68a73dcea11<br>Case study 2: https://figshare.com/s/5dcefa52f529ad34dc02<br>Case study 3: https://figshare.com/s/2c5fb050f2d2b9e01170

For manuscripts utilizing custom algorithms or software that are central to the research but not yet described in published literature, software must be made available to editors and reviewers. We strongly encourage code deposition in a community repository (e.g. GitHub). See the Nature Portfolio guidelines for submitting code & software for further information.

Data

Policy information about availability of data

All manuscripts must include a data availability statement. This statement should provide the following information, where applicable:

- Accession codes, unique identifiers, or web links for publicly available datasets
- A description of any restrictions on data availability
- For clinical datasets or third party data, please ensure that the statement adheres to our policy

• Case study 1 (North Sea fishes):
- Abundance data are available at:
https://datras.ices.dk/Data_products/Download/Download_Data_public.aspx (source data)
and at:
https://figshare.com/s/9d3cd1d6f68a73dcea11 (input table for analyses).
- Traits data are available through the PANGAEA traits database at:
https://doi.org/10.1594/PANGAEA.900866 (source data).

• Case study 2 (Marine mammals):
- Presence/absence and traits data are available at:
https://figshare.com/s/5dcefa52f529ad34dc02
- Range maps data are available at : http://www.iucnredlist.org

• Case study 3 (Reef fishes):
- Forecasted habitat suitability data and associated traits can be made available from the authors upon reasonable request.
- Traits data are made available at : https://figshare.com/s/2c5fb050f2d2b9e01170
- GASPAR (Traits data and occurrences can be made available from the authors upon reasonable request.)
- SERF (Occurrences can be made available from the authors upon reasonable request)
- Global occurrence data from GBIF are available at: https://www.gbif.org/
- Global occurrence data from OBIS are available at: https://obis.org/
- Reef Life Survey data are available at: https://reeflifesurvey.com/survey-data/

• Projection of environmental variables from the CMIP6 Earth system model are available at: https://esgf-node.llnl.gov/projects/cmip6/.

Field-specific reporting

Please select the one below that is the best fit for your research. If you are not sure, read the appropriate sections before making your selection.

☐ Life sciences ☐ Behavioural & social sciences ☒ Ecological, evolutionary & environmental sciences

For a reference copy of the document with all sections, see nature.com/documents/nr-reporting-summary-flat.pdf

Ecological, evolutionary & environmental sciences study design

All studies must disclose on these points even when the disclosure is negative.

Study description	This study introduces a novel framework for quantifying the vulnerability of biological communities by integrating the precautionary principle and ecological traits. There is indeed a need to prioritize the vulnerability of biological communities by considering the uncertainty in how human and environmental threats will change and how species will respond to them. Here we address these uncertainties by simulating community potential response to the widest range of threats. Our framework is applicable on a wide variety of data (abundance, presence/absence and probability of presence), spatial scale (from local to global) and time period (past, present, future). We applied our framework to three complementary cases: i) the vulnerability trend in North Sea fishes in the past four decades, ii) present-day spatial vulnerability of global marine mammals, and iii) projected vulnerability changes in global reef fishes under climate change scenario.
Research sample	i) The abundance of North Sea fishes species come from bottom trawl survey (NS-IBTS) and data are available at: https://datras.ices.dk/Data_products/Download/Download_Data_public.aspx (source data) and at: https://figshare.com/s/b229e52cce6da45b75f2 (input table for vulnerability analyses).<br><br>ii) We selected a representative data set currently used in macro-ecology at global scale. This sample encompass the presence absence of almost all marine mammals and the associated traits data for these species (case study 2). All the data are available at: https://figshare.com/articles/Input_data_for_Global_vulnerability_of_marine_mammals_to_global_warming_/11323304 and at: http://www.iucnredlist.org for species range maps.<br><br>iii) We compile a novel point record dataset on reef species occurrence and biomass available globally which provided a highly

	comprehensive spatial coverage of reef species distributions and thus was an appropriate choice for our study. Our final set comprised of 2,340 species at 496,309 unique georeferenced locations in 20,450 grid cells for analyses. We combined together presence/absence data obtained from OBIS and GBIF with in-hand reef fish underwater visual census observations. The in-hand data come from curated citizen science and professional surveys, namely the Reef Life Survey (RLS, Edgar & Stuart-Smith 2014), Socio-Ecological Reef Fish dataset (SERF, Mora et al. 2011, Cinner et al. 2018) and GASPAR project dataset (see supporting information in Barneche et al. 2018 for full sampling description). We used these data to derived habitat suitability models (case study 3) which can be made available from the authors upon reasonable request.
Sampling strategy	- Case study 1 (North Sea fishes):<br>Sampling in a regular grid (protocol available here: https://www.ices.dk/sites/pub/Publication%20Reports/ICES%20Survey%20Protocols%20(SISP)/SISP%2010%20%E2%80%93%20Revision%2011_Manual%20for%20the%20North%20Sea%20International%20Bottom%20Trawl%20Surveys.pdf).<br><br>- Case study 2 (Marine mammals):<br>We compiled geographic range maps from the IUCN database (http://www.iucnredlist.org) for all known marine mammal species (127 species). We deleted 5 species for which a major part of their range fall in freshwater environment (Sotalia Fluviatili, Inia geoffrensis Platanista gangetica, Pusa sibirica and Trichechus inunguis) and we finally retained 122 species. We then established a presence/absence matrix and derived SR by overlapping the geographic ranges and counting how many species occur in each grid cell (1° × 1° grid cells, ~10,000 km²).<br><br>- Case study 3 (Reef fishes): All species distribution models used in case study 3 had a minimum of 50 occurrences which is a conservative lower limit for this statistical approach, as determined by the following articles https://doi.org/10.1111/ecog.01509; https://doi.org/10.1111/j.1472-4642.2008.00482.x.
Data collection	- Case study 1 (North Sea fishes):<br>Abundance data were collected thanks to a many persons on oceanographic vessels that catched demersal fish communities with vertical a bottom trawl. After each haul, fishes were identify at species level and counted. Corresponding data were then uploaded on the DATRAS repository platform (https://datras.ices.dk/Data_products/Download/Download_Data_public.aspx). The first author (Arnaud Auber) extracted the data from the DATRAS database. Traits data were obtained through accumulation of observations and measurements during the last decades in various studies. These data were then combined and uploaded in the PANGAEA traits database thanks to Beukhof et al., 2019, at: https://doi.pangaea.de/10.1594/PANGAEA.900866.<br><br>- Case study 2 (Marine mammals):<br>Considering the marine mammals data set, we downloaded the data from the IUCN database (http://www.iucnredlist.org). We conducted all the analyses by using the software R.<br><br>- Case study 3 (Reef fishes): Data were collected by professional and trained scuba divers recording fishes species identities and body size observed on underwater visual transects and fish body sizes (Reaf Life Survey, SERF, GASPAR), this is a well-established method for estimated fish community structure (see https://www.nature.com/articles/sdata20147; https://doi.org/10.1016/j.biocon.2020.108855)
Timing and spatial scale	- Case study 1 (North Sea fishes):<br>Abundance data from 1983 to 2019 were selected at the North Sea scale. The starting year (1983) was selected based upon field experts who identified 1983 as the first year where the sampling protocol was considered as constant. Because the NS-IBTS survey is mainly funded to assess fish stocks, and thus to define annual fishing quotas, the sampling is done once a year (during the first quarter). The last year (2019) was selected as the last year of our temporal window because 2020 data were not still available when we extracted data. The spatial scale of the North Sea was used in order to have only one vulnerability value per year for the entire ecosystem.<br><br>- Case study 2 (Marine mammals):<br>We used the marine mammals traits collated by Albouy et al 2017 and the IUCN shapefile for marine mammals (version 3) at global scale download from the IUCN website in February 2016. These data span on several decades with opportunistic observations to present day at a global scale.There is therfore no periodicity since the database only contains opportunistic observations.<br><br>- Case study 3 (Reef fishes):<br>Reef fish occurrences were compiled by the Global Biodiversity Information Facility and Ocean Biodiversity Information System which provide access to unstructured biodiversity observations. These data span 1990 to present day at a global scale.
Data exclusions	Sites for which species richness was inferior to 10 were not considered to prevent any over or underestimation of the functional vulnerability (case study 3).
Reproducibility	R codes were computed in a way to be reproducible by users. Explanations are within the provided code
Randomization	No randomization was required because this is a study using observational data.
Blinding	This is not an experimental design, so no blinding was required in this study.

Did the study involve field work? ☒ Yes ☐ No

Field work, collection and transport

Field conditions	Sampling by bottom traw survey and scuba diving in varying weather conditions
Location	Sampling in the North Sea, and at global scale for reef fish communities data (scuba)
Access & import/export	For each survey, every year, a trawling permit was obtained from local authorities to collect the samples.
Disturbance	No disturbance

Reporting for specific materials, systems and methods

We require information from authors about some types of materials, experimental systems and methods used in many studies. Here, indicate whether each material, system or method listed is relevant to your study. If you are not sure if a list item applies to your research, read the appropriate section before selecting a response.

Materials & experimental systems

n/a	Involved in the study	
☒	☐	Antibodies
☒	☐	Eukaryotic cell lines
☒	☐	Palaeontology and archaeology
☐	☒	Animals and other organisms
☒	☐	Human research participants
☒	☐	Clinical data
☒	☐	Dual use research of concern

Methods

n/a	Involved in the study	
☒	☐	ChIP-seq
☒	☐	Flow cytometry
☒	☐	MRI-based neuroimaging

Animals and other organisms

Policy information about studies involving animals; ARRIVE guidelines recommended for reporting animal research

Laboratory animals	This study didn't involve laboratory animal
Wild animals	For the North Sea fish survey, fishes were caught through 30 min trawling hauls. The NS-IBTS survey is an European Survey designed to assess fish stocks of exploited fish species (these stocks informations are then used to detrmine quotas that fishermen must respect). These trawlings are performed thanks to trawling permits themselves obtained after requests to local authorities every years by France, UK, Germany, Netherland, Sweden, Norway and Denmark. No invasive practices with the other data acquisition (i.e., scuba diving).
Field-collected samples	No laboratory work
Ethics oversight	For the NS-IBTS survey (i.e., North Sea), trawling authorizations are provided by each of the following countries : UK, France, Germany, Netherlands, Sweden, Norway, Denmark.

Note that full information on the approval of the study protocol must also be provided in the manuscript.
